# Association Between Physical Activity and Odds of Chronic Conditions Among Workers in Spain

**DOI:** 10.5888/pcd17.200105

**Published:** 2020-10-08

**Authors:** Rubén López-Bueno, Rúni Bláfoss, Joaquín Calatayud, Guillermo F. López-Sánchez, Lee Smith, Lars L. Andersen, José A. Casajús

**Affiliations:** 1 Department of Physical Medicine and Nursing, University of Zaragoza, Zaragoza, Spain; 2 Musculoskeletal Disorders and Physical Workload, National Research Centre for the Working Environment, Copenhagen, Denmark; 3 Department of Sports Science and Clinical Biomechanics, Muscle Research Cluster, University of Southern Denmark, Odense, Denmark; 4 Exercise Intervention for Health Research Group (EXINH-RG), Department of Physiotherapy, University of Valencia, Valencia, Spain; 5 Faculty of Sport Sciences, University of Murcia, Murcia, Spain; 6 The Cambridge Centre for Sports and Exercise Sciences, Anglia Ruskin University, Cambridge, United Kingdom; 7 Department of Health Science and Technology, Aalborg University, Aalborg, Denmark; 8 Faculty of Health Sciences, University of Zaragoza, Zaragoza, Spain; 9 Growth, Exercise, Nutrition and Development Research Group, University of Zaragoza, Zaragoza, Spain; 10 Biomedical Research Networking Centre about Nutrition and Obesity Physiopathology (CIBER-OBN), Madrid, Spain

## Abstract

**Introduction:**

Prevention of chronic conditions is a major public health challenge, and achieving minimum recommended levels of physical activity aids in reaching this objective. The aim of our study was to investigate whether levels of physical activity were associated with the prevalence of common chronic conditions among the Spanish workforce.

**Methods:**

We retrieved data from the Spanish National Health Survey 2017 (N = 9,695) in which the mean age of participants was 44.4 (standard deviation, 10.4 y), and 47.4% were women. Workers self-reported a set of 6 chronic conditions (ie, chronic low-back pain, chronic neck pain, diabetes, hypertension, depression, and anxiety), and we used the International Physical Activity Questionnaire short form to estimate physical activity. We performed multivariable logistic regression adjusted for possible confounders to assess associations between physical activity and chronic conditions.

**Results:**

The final adjusted model showed that performing less than 600 metabolic equivalent-minutes per week of physical activity was associated with significantly increased odds for chronic conditions (adjusted odds ratio [aOR] = 1.18; 95% CI, 1.07–1.30). Of the sex and age subgroups analyzed, this association was significant in men aged 17 to 44 (aOR = 1.21; 95% CI, 1.00–1.46). Among chronic conditions, low-back pain and anxiety were associated with low levels of physical activity, whereas covariates such as body mass index, smoking habits, education level, and occupational class had an important influence on the association between physical activity and chronic conditions.

**Conclusion:**

Results suggest that achieving sufficient physical activity could reduce chronic conditions among Spanish workers.

**Summary**

**What is already known on this topic?**

High-quality evidence indicates that physical activity reduces the risk of the most prevalent chronic conditions.

**What is added by this report?**

Insufficient weekly physical activity is associated with increased odds of chronic conditions among a Spanish population of workers.

**What are the implications for public health practice?**

Physical activity strategies for meeting recommended guidelines might reduce levels of some of the most prevalent chronic conditions among the Spanish workforce.

## Introduction

A key indicator related to the health status of a country is the health of its workforce. A high prevalence of chronic diseases and conditions in a working population can lead to a set of undesirable consequences ranging from sickness absenteeism to reduced productivity and a rise in disability pensions (1,2). In addition, the national financial cost due to sickness absence related to chronic musculoskeletal disorders in countries such as Holland amounted to €1.3 billion annually (3); thus, acquiring knowledge related to health behaviors of workers can aid in the implementation of successful strategies to improve health and reduce related costs. Chronic diseases are the leading cause of death among the Spanish population, whereas chronic conditions such as low-back pain and neck pain are the primary reasons for disability-adjusted life years (4). Healthy habits such as regular physical activity are associated with an increased lifespan; for example, combined work and household physical activity have been observed to reduce mortality risk in Spanish men and women, whereas similar mortality risk reductions for leisure-time physical activity have been observed only in Spanish women (5). Such distinction among physical activity domains as well as between the sexes is noteworthy, because physical activity during leisure time is usually linked to lower mortality risk in both men and women, and high levels of occupational physical activity have been linked to increased mortality risk in men (6,7). Furthermore, high leisure-time physical activity was associated with low prevalence of hypertension, diabetes, hypercholesterolemia, depression, and anxiety and reductions in the use of prescription medication in a dose–response fashion among a general population of Spanish adults (8). Contrarily, occupational physical activity has been observed to increase both the risk for disability pension and the risk for long-term sickness absence (9,10).

The World Health Organization (WHO) recommends that adults perform at least 150 minutes of moderate-intensity physical activity, or 75 minutes of vigorous-intensity physical activity, or an equivalent combination of moderate- and vigorous-intensity physical activity to achieve at least 600 MET-minutes per week (MET = metabolic equivalent of task, ie, a caloric expenditure unit) (11). Because increased physical activity has been associated with reduced prevalence of chronic conditions among general populations of adults and among workers from other countries (8,12), it is reasonable to expect that meeting recommended physical activity guidelines would be associated with reduced prevalence of several of the most common chronic diseases and conditions among the Spanish workforce. A recent study by López-Sánchez et al (13) estimated that 30.2% of the Spanish population is not meeting current international guidelines for weekly physical activity, suggesting that insufficient physical activity will also be a critical issue related to chronic conditions among the general working population. Furthermore, such physical activity levels could substantially vary depending on sex and age (ie, men engage in more vigorous and light physical activity overall, whereas women perform more moderate physical activity; also, adults aged 18–65 years perform more moderate-to-vigorous physical activity than adolescents aged 13-17 and seniors aged 65 to 75 to achieve physical activity guidelines) (14). Because most studies involving workers and health habits are of populations from countries other than Spain, little is known about how physical activity may affect chronic conditions among Spanish workers where working conditions and lifestyle are different (15,16). Thus, our study aimed to investigate the association between physical activity and chronic conditions among Spanish workers. We hypothesized an inverse association between physical activity and a set of the most prevalent chronic conditions in the workforce.

## Methods

### Study design and population

We retrieved data from the Spanish National Health Survey 2017 (ENSE 2017), a regular survey assessing general health among Spanish children and adults every 5 years (17). Data collection was carried out through the survey, which was conducted in Spain from October 2016 through October 2017 by the Ministry of Health, Social Services, and Equality and the National Statistics Institute; anonymized data series from the current and previous rounds of the survey are publicly available from an institutional web server (17). A computer-assisted personal interview was conducted in the homes of selected participants, who were assisted by trained interviewers. We implemented a stratified 3-stage sampling that considered census sections, family households, and participants aged ≥15. Households were selected by using systematic sampling, and the random Kish method, which assigns equal probability to all potential participants in the household, was used to select the household participant to complete the questionnaire (18). The sample was distributed throughout all Spanish regions assigning both a uniform part and other variable parts according to proportional regional size and accounting for studied characteristics, type of respondent, and information from other surveys (19). Sections were selected within each stratum with probability proportional to their size. In each section, households were selected with equal probability by systematic sampling. This procedure led to self-weighting samples in each stratum. 

The original sample comprised 37,500 households distributed in 2,500 sections, in which 30.1% (n = 11,287) of the selected households did not reply to the survey for several reasons (ie, absence, empty dwelling, refusal or inability to answer). As a result, a representative sample of the Spanish adult population comprising 23,089 participants aged 15 to 103 (a survey response rate of 69.9%) was collected.

Because the International Physical Activity Questionnaire (IPAQ) short form was not included in the ENSE 2017 questionnaires for participants aged ≥70, those participants were excluded from our study analyses (n = 5,310). Of the remaining population, those below the legal working age (<16) and unemployed participants were also excluded (n = 7,894). Overall, data from 22 survey questions were retrieved for this study: 7 questions regarding physical activity; 1 question each regarding age, sex, height, weight, education level, occupational class, occupational physical activity, smoking status, and fruit consumption; and 6 regarding chronic conditions. In addition, those remaining participants with missing values in any of the study variables (n = 190) were also removed from statistical analyses. Therefore, our study consisted of a total of 9,695 participants from a general working population.

Data were reported in adherence with Strengthening the Reporting of Observational Studies in Epidemiology (STROBE) guidelines (20). All participants gave signed consent before completing the survey.

### Chronic condition (outcome)

We identified 6 of the most prevalent chronic conditions in the Spanish workforce (1). Measures on chronic conditions were obtained through the following question: “Have you suffered from hypertension within the last 12 months?” Participants answering “yes” to this question were considered to have experienced hypertension during that period. The same procedure was used for assessing the prevalence of the remaining chronic conditions from the study set (ie, diabetes, chronic neck pain, chronic low-back pain, depression, and anxiety). Finally, we created an outcome variable concerning the experience of having had at least 1 of the mentioned chronic conditions; those participants answering “yes” to having 1 or more chronic conditions were included in the chronic condition category.

### Physical activity (exposure)

The questions of the IPAQ short version questions embedded in the healthy habits section of ENSE 2017 were used to estimate physical activity (21). IPAQ has been shown to provide a valid and reliable physical activity estimation when tested in different adult populations worldwide, presenting sufficient validity (Spearman´s ρ = 0.30; 95% CI, 0.23–0.36) and reliability (Spearman’s ρ = 0.81; 95% CI, 0.79–0.82) (21). Overall physical activity MET minutes per week were estimated as the sum of vigorous + moderate + walking MET-minutes-per-week scores (22). Following the analysis protocol of IPAQ, physical activity was categorized into METs according to WHO guidelines: 1) <600 MET-minutes/week and 2) ≥600 MET-minutes per week (22).

### Covariates

According to prior work, our study controlled for sociodemographic factors (age, sex, education level, and occupational class), and lifestyle factors (body mass index, occupational physical activity, fruit consumption, and smoking status) (23–26).

Education level was divided into 3 categories indicating the highest educational achievement (ie, completion of primary or lower school, secondary school, or tertiary [college degree]). We categorized 168 different occupational groups into 6 groups by using the Spanish national list of occupations (27): occupational class I, executive managers and academics; occupational class II, middle managers, technicians, athletes, and artists; occupational class III, white-collar and self-employed workers; occupational class IV, supervisors and skilled blue-collar workers from the secondary economic sector (ie, manufacturing, engineering, and construction); occupational class V, skilled blue-collar workers from the primary economic sector (ie, agriculture, mining, and other natural resource industries); and occupational class VI, unskilled workers.

Body mass index (BMI) was derived from self-reported height and weight and calculated as weight in kilograms divided by height in squared meters with categories set according to WHO guidelines (obese, BMI ≥30 kg/m^2^; overweight, BMI ≥25 kg/m^2^; normal, BMI 18.5–24.9 kg/m^2^; and underweight, BMI <18.5 kg/m^2^). Fruit consumption was assessed on the basis of findings from a study of industrial workers and cardiovascular risk factors and was divided into 2 groups: people who consumed at least 1 piece of fresh fruit (excluding juices) a week, and those who did not (28).

Occupational physical activity was estimated by asking the following question: “Which of the following better describes your main activity during the working hours?” Possible answers were: “sit most of the working hours” (sedentary), “standing up most of the working hours without performing high efforts” (low), “walking, carrying any weight, with frequent displacements” (moderate), and “performing high physically demanding tasks” (high). Finally, smoking status was categorized as “current smoker,” “former smoker,” and “never smoker.”

### Statistical analyses

We used SPSS 22.0 (IBM Corp) to perform statistical analyses. Differences in the prevalence of chronic conditions among subgroups within each variable were assessed by χ^2^ tests. The association between physical activity (exposure) and chronic condition (outcome) was estimated through multivariable logistic regression analysis conducted for both the whole sample and by sex and age subgroups. To ensure sufficient statistical power when comparing groups by age, the mean age of the sample was set as the cut-off point. Sex- and age-segmented analyses were adjusted for education level, occupational class, occupational physical activity, BMI, fruit consumption, and smoking status, whereas the analysis for the overall sample was also adjusted for sex and age. All covariates were included in the models as categorical variables. Associations were calculated between having 1 or more of the 6 chronic conditions examined and physical activity and between each of the chronic conditions and physical activity.

Participants with missing data (2%) were not included in the analyses. We used logistic regression analyses to calculate adjusted odds ratios (aORs) with 95% CIs. The level of significance was set at *P* < .05.

## Results

Our study sample of 9,695 comprised a general population of Spanish workers (52.6% men) with a mean age of 44.4 years (SD, 10.4) (Table 1). Most workers (69.6%) reached WHO guidelines for adequate weekly physical activity (≥600 MET-minutes/week). We found significant differences among subgroups in relation to prevalence of chronic conditions for most of the covariates: age, sex, BMI, smoking status, education level, occupational class, and occupational physical activity.

**Table 1:** Participant (N = 9,695) Characteristics, 2017 Spanish National Health Survey

Characteristic	Total sample, n (%)	Has one or more chronic condition^a^	
		n (%)	*P* Value^b^
**Age, y**			
17–44	4,915 (50.7)	998 (20.3)	< .001
45–69	4,780 (49.3)	2,127 (44.5)	
**Sex**			
Men	5,100 (52.6)	1,520 (29.8)	< .001
Women	4,595 (47.4)	1,604 (34.9)	
**Body mass index (weight in kilograms/height in meters^2^)^c^**			
Underweight	180 (1.9)	45 (25.0)	< .001
Normal	4,556 (47.0)	1,189 (26.1)	
Overweight	3,600 (37.1)	1,274 (35.4)	
Obese	1,359 (14.0)	612 (45.0)	
**Smoking status**			
Never smoked	4,188 (43.2)	1,160 (27.7)	< .001
Former smoker	2,608 (26.9)	996 (38.2)	
Smoker	2,899 (29.9)	861 (29.7)	
**Education level^d^**			
≤Primary	950 (9.8)	433 (45.6)	< .001
Secondary	5,831 (60.1)	1,977 (33.9)	
≥Tertiary	2,914 (30.1.)	723 (24.8)	
**Occupational class^e^**			
I	1,299 (13.4)	291 (22.4)	< .001
II	979 (10.1)	232 (23.7)	
III	2,210 (22.8)	674 (30.5)	
IV	1,018 (10.5)	307 (30.2)	
V	2,870 (29.6)	935 (32.8)	
VI	1,319 (13.6)	454 (34.4)	
**Physicial activity, MET minutes per wk^f^**			
≥ 600	6,748 (69.6)	2,045 (30.3)	< .001
< 600	2,947 (30.4)	1,079 (36.6)	
**Consumed ≥1 pieces of fruit per week**			
Yes	9,172 (94.6)	2,935 (32.0)	.40
No	523 (5.4)	176 (33.7)	
**Occupational physical activity^g^**			
Sedentary	3,180 (32.8)	967 (30.4)	< .05
Low	4,179 (43.1)	1,362 (32.6)	
Moderate	1,812 (18.7)	607 (33.5)	
High	524 (5.4)	184 (35.1)	

a Hypertension, diabetes, chronic neck pain, chronic low-back pain, depression, and anxiety.

b Calculated by χ^2^ test.

c BMI = weight in kilograms divided by height in squared meters with categories set according to World Health Organization guidelines (obese, BMI ≥30 kg/m^2^; overweight, BMI ≥25 kg/m^2^; normal, BMI 18.5–24.9 kg/m^2^; and underweight, BMI <18.5 kg/m^2^).

d Primary (grades 1–6); secondary (grades 7–12); tertiary (college degree).

e Occupational class I (executive managers and academics); occupational class II (middle managers, technicians, athletes, and artists); occupational class III (white-collar and self-employed workers); occupational class IV (supervisors and skilled blue-collar workers from the secondary economic sector [ie, manufacturing, engineering, and construction]); occupational class V (skilled blue-collar workers from the primary economic sector [ie, agriculture, mining, and other natural resource industries]); occupational class VI (unskilled workers).

f MET = Metabolic Equivalent of Task (ie, a caloric expenditure unit).

g Sit most of the working hours (sedentary); stand up most of the working hours without performing high efforts (low); walking, carrying any weight, with frequent displacements (moderate); and performing high physically demanding tasks (high).

The final adjusted model on the entire study sample showed that less than 600 MET-minutes per week of physical activity was associated with significantly increased odds for a chronic condition (aOR = 1.18; 95% CI, 1.07–1.30) (Table 2). Women, older age, former and current smoker, primary and secondary education only, obesity and overweight condition, and no weekly fruit consumption subgroups were also associated with significantly higher odds for chronic conditions compared with their reference in each covariate, whereas the contrary, significantly lower odds for chronic conditions, occurred with the occupational class subgroup I when compared with its reference (Table 2).

**Table 2:** Association of Physical Activity and Covariates With Chronic Conditions^a^ (Outcome) Estimated by Multivariable Logistic Regression, Participants (N = 9,695), 2017 Spanish National Health Survey

Characteristic	Has One or More Chronic Conditions^b^
**Physical activity, MET-minutes per week^c^**	
<600	1.18 (1.07–1.30)^d^
≥600	1[Reference]
**Age, y**	
45–69	2.92 (2.65–3.21)^e^
17–44	1[Reference]
**Sex**	
Women	1.59 (1.44–1.76)^e^
Men	1[Reference]
**Education level^f^**	
≤ Primary	1.55 (1.27–1.90)^e^
Secondary	1.24 (1.07–1.43)^e^
≥ Tertiary	1[Reference]
**Smoking status**	
Current	1.20 (1.08–1.35)^e^
Former	1.29 (1.16–1.45)^e^
Never	1[Reference]
**Body mass index** **(BMI)^g^**	
Obese	2.13 (1.45–3.12)^e^
Overweight	1.51 (1.04–2.19)^e^
Normal	1.05 (0.73–1.52)
Low	1[Reference]
**Occupational class^h^**	
I	0.71 (0.56–0.89)^i^
II	0.94 (0.76–1.18)
III	0.91 (0.77–1.08)
IV	1.02 (0.84–1.24)
V	1.05 (0.91–1.22)
VI	1[Reference]
**Consumed ≥1 pieces of fruit per week**	
No	1.23 (1.00–1.50)^e^
Yes	1[Reference]
**Occupational physical activity^j^**	
Sedentary	0.94 (0.75–1.18)
Low	0.86 (0.70–1.07)
Moderate	0.85 (0.68–1.06)
High	1[Reference]

a Hypertension, diabetes, chronic neck pain, chronic low-back pain, depression, or anxiety.

b Values are adjusted odds ratios (95% CI). Each variable has been adjusted for the other variables.

c MET = Metabolic Equivalent of Task (ie, a caloric expenditure unit).

d Significant at *P* < .05.

e Significant at *P* < .001.

f Primary (grades 1–6); secondary (grades 7–12); tertiary (college degree).

g BMI = weight in kilograms divided by height in squared meters with categories set according to World Health Organization guidelines (obese, BMI ≥30 kg/m^2^; overweight, BMI ≥25 kg/m^2^; normal, BMI 18.5–24.9 kg/m^2^; and underweight, BMI <18.5 kg/m^2^).

h Occupational class I (executive managers and academics); occupational class II (middle managers, technicians, athletes, and artists); occupational class III (white-collar and self-employed workers); occupational class IV (supervisors and skilled blue-collar workers from the secondary economic sector [ie, manufacturing, engineering, and construction]); occupational class V (skilled blue-collar workers from the primary economic sector [ie, agriculture, mining, and other natural resource industries]); occupational class VI (unskilled workers).

i Significant at *P* < .01.

j Sit most of the working hours (sedentary); stand up most of the working hours without performing high efforts (low); walking, carrying any weight, with frequent displacements (moderate); and performing high physically demanding tasks (high).

In sex- and age-segmented analyses and adjusted analyses (Table 3), the association between physical activity and chronic conditions remained significant among the subgroup formed by men aged 17 to 44 (aOR = 1.21; 95% CI, 1.00–1.46).

**Table 3:** Association Between <600 MET-Minutes of Physical Activity per Week and Chronic Conditions, Estimated By Multivariable Logistic Regression, Participants (N = 9,695), 2017 Spanish National Health Survey^a^

Sex	Age, Years	Adjusted Odds Ratio (95% CI)
Men	17–44	1.21 (1.00–1.46)^b^
	45–69	1.21 (0.98–1.48)
Women	17–44	1.11 (0.93–1.33)
	45–69	1.22 (0.96–1.56)

Abbreviation: MET, metabolic equivalent of task.

a Adjusted for education level, body mass index (weight in kilograms divided by height in square meters), smoking status (never smoked, former smoker, current smoker), occupational class (I [executive managers and academics], II [middle managers, technicians, athletes and artists], III [white-collar and self-employed workers], IV [supervisors and skilled blue-collar workers from the secondary economic sector: manufacturing, engineering, and construction], V [skilled blue-collar workers from the primary economic sector: agriculture, mining, and other natural resource industries], VI [unskilled workers]), fruit consumption (whether consumed 1 piece of fruit or more per week), and occupational physical activity (sit most of the working hour (sedentary); stand up most of the working hours without performing high efforts (low); walking, carrying any weight, with frequent displacements (moderate); and performing high physically demanding tasks (high).

b Significant at *P* < .05.

Finally, an analysis of associations between physical activity and each of the examined chronic conditions showed that achieving recommended physical activity guidelines was associated with significantly lower odds for low-back pain (aOR = 0.80; 95% CI, 0.70–0.91) and anxiety (aOR = 0.67; 95% CI, 0.54–0.84) (Table 4).

**Table 4:** Association Between Physical Activity Levels and Selected Chronic Diseases, Estimated by Multivariable Logistic Regression, Participants (N = 9,695), 2017 Spanish National Health Survey^a ^

Chronic Disease	aOR (95% CI)
Hypertension	0.89 (0.76–1.03)
Diabetes	1.03 (0.79–1.34)
Low-back pain	0.80 (0.70–0.91)^b^
Neck pain	0.97 (0.83–1.14)
Anxiety	0.67 (0.54–0.84)^c^
Depression	1.10 (0.84–1.44)

Abbreviation: aOR, adjusted odds ratio.

a Association between adequate physical activity (≥600 MET-minutes/week) and chronic disease. Adjusted for sex, age, education, body mass index, smoking, occupational class, fruit consumption, and occupational physical activity.

b Significant at *P* < .01.

c Significant at *P* < .001.

## Discussion

Our study suggests that performing less than 600 MET-minutes per week of physical activity is associated with significantly increased odds for chronic conditions among a general population of Spanish workers. When stratified by sex and age, men aged 17 to 44 years not achieving 600 MET-minutes per week had higher odds of chronic conditions than other subgroups. In addition, participants reporting less than 600 MET-minutes per week had significantly higher odds for experiencing low-back pain and anxiety than their 600 MET-minutes per week or more counterparts. These results are consistent with our hypothesis and with previous research that observed significant associations between low physical activity and chronic conditions among a Spanish adult population (8). Our study adds to the existing literature about the importance of meeting WHO physical activity guidelines to prevent some of the most prevalent chronic conditions usually experienced by working populations.

Because chronic conditions have been identified as major causes of sickness absenteeism and disability pension (29,30), strategies should be implemented to reduce their current incidence. Our study suggests that performing the recommended amount of physical activity could be a way forward. Both sickness absenteeism and disability pension entail a substantial economic cost to society (31) and constitute a public health issue (32,33).

Interestingly, findings from our study also suggest that workers aged 45 to 69 not meeting current physical activity guidelines are more likely to experience chronic conditions that their younger counterparts. The aging process itself is usually related to an increased probability of experiencing chronic conditions (34); however, as observed in our results, healthy habits such as physical activity could reduce the prevalence of chronic conditions among older workers. As suggested by Brawner et al (12), this association may be bidirectional, because chronic conditions among older adults have been associated with low physical activity levels. Our study found that physical activity was significantly linked with reduced prevalence of low-back pain and anxiety, which supports the results of previous studies specifically focused on these conditions; a meta-analysis of prospective cohort studies found risk of chronic low-back pain was reduced by 11% to 16% with leisure-time physical activity, and other observational studies found that meeting physical activity guidelines was associated with 13.5% reduced odds of anxiety (35,36).

Although our study did not differentiate among physical activity domains, previous research has underscored the benefits of both leisure-time and commuting physical activity, whereas the opposite has been observed for occupational physical activity (9,37). Thus, because differences in physical activity levels and domains have been observed among European countries (15), generalizations about working populations from other countries might be mediated by these differences. Furthermore, the strong influence that both higher occupational class and lower education level had on the relationship between physical activity and chronic conditions suggests that other socioeconomic characteristics could also exacerbate these variations; previous research has already indicated the strong association between physical activity and both occupational class and education level (ie, those from lower occupational classes and education levels tend to engage in less physical activity and have worse health-related habits, and consequently are more likely to suffer from chronic conditions). (38,39).

The strengths of our study are its use of a large representative sample and its estimating physical activity through a validated tool. In addition, we used a broad range of covariates to control for the relationship between exposure and outcome variables. Nevertheless, for a better interpretation of the results, several limitations should be considered. First, because answers may have been influenced by common method variance, in which a person’s mood or disease status affected answers, the possibility of recall bias exists. Second, the IPAQ short form does not differentiate among physical activity domains. Such information could clarify why physical activity has beneficial properties relative to chronic conditions. Last, the cross-sectional study design did not allow causal interpretations; for instance, several chronic conditions may reduce participation in physical activity. Thus reduced physical activity might not be the cause of a chronic condition, but the consequence.

In conclusion, the results of our study suggest that meeting WHO physical activity guidelines might be essential to reduce chronic conditions among the general workforce. Older workers in particular and those experiencing either low-back pain or anxiety were observed to be the most adversely influenced by insufficient physical activity; thus, strategies aimed at older workers could be critical to prevent them from experiencing chronic conditions.
